# Supplementary figures and images for: Statistical prediction of microbial metabolic traits from genomes
Source: PLoS Comput Biol. 2023 Dec 19;19(12):e1011705. doi: 10.1371/journal.pcbi.1011705 (PMC10729968; doi:10.1371/journal.pcbi.1011705)

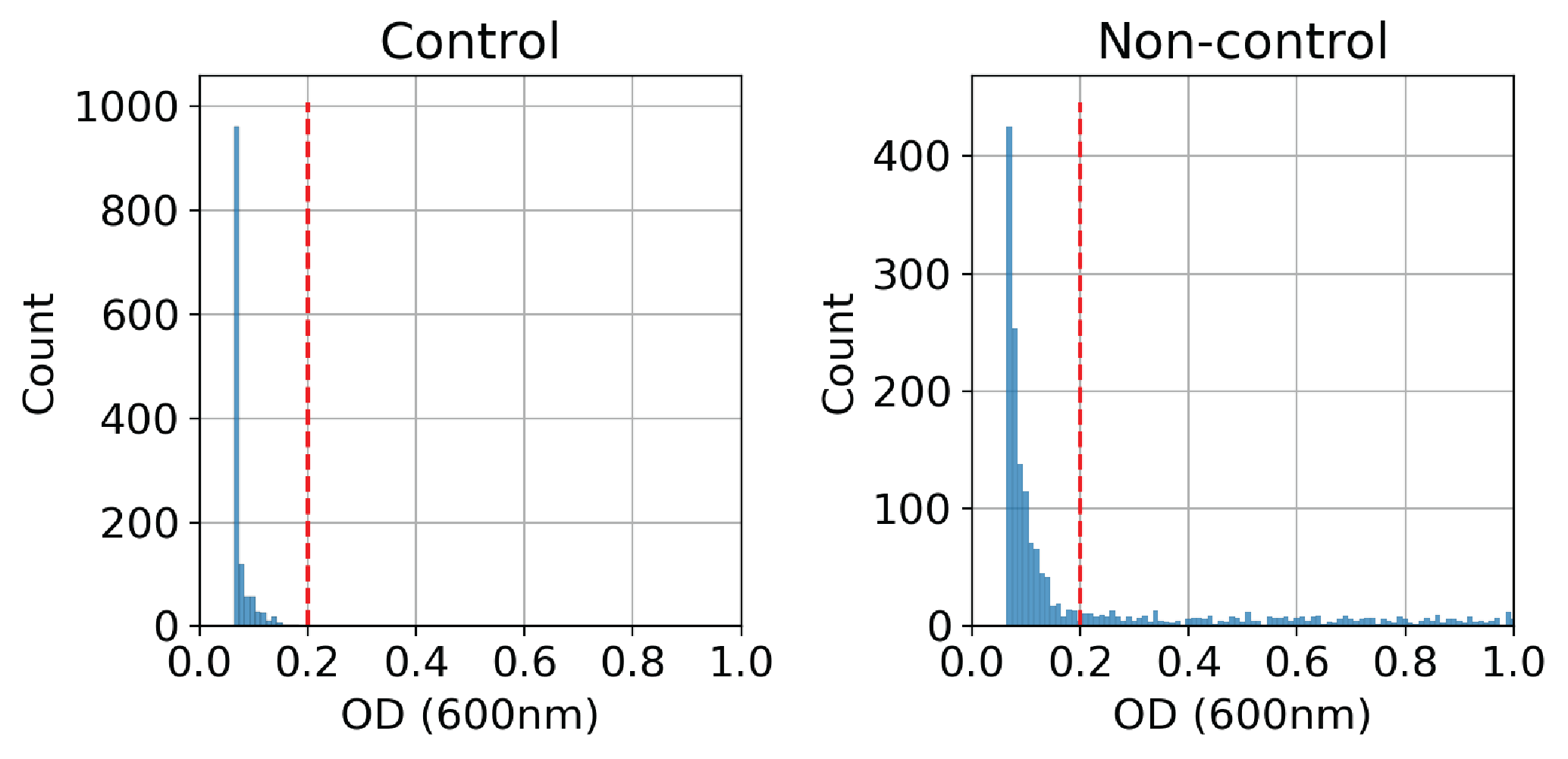

Supplement: S1 Fig — Distribution of OD (600nm) measurements after 72-hours of incubation. The left panel shows the OD values of control groups, including wells with either no carbon source in the medium or no inoculated culture. The right panel shows OD of experimental groups, where wash cell cultures are inoculated to media with a carbon source. The red dashed line indicates the growth/no-growth threshold (0.2), where we observed a clear cutoff of the control group wells. (TIF) [file pcbi.1011705.s001.tif]

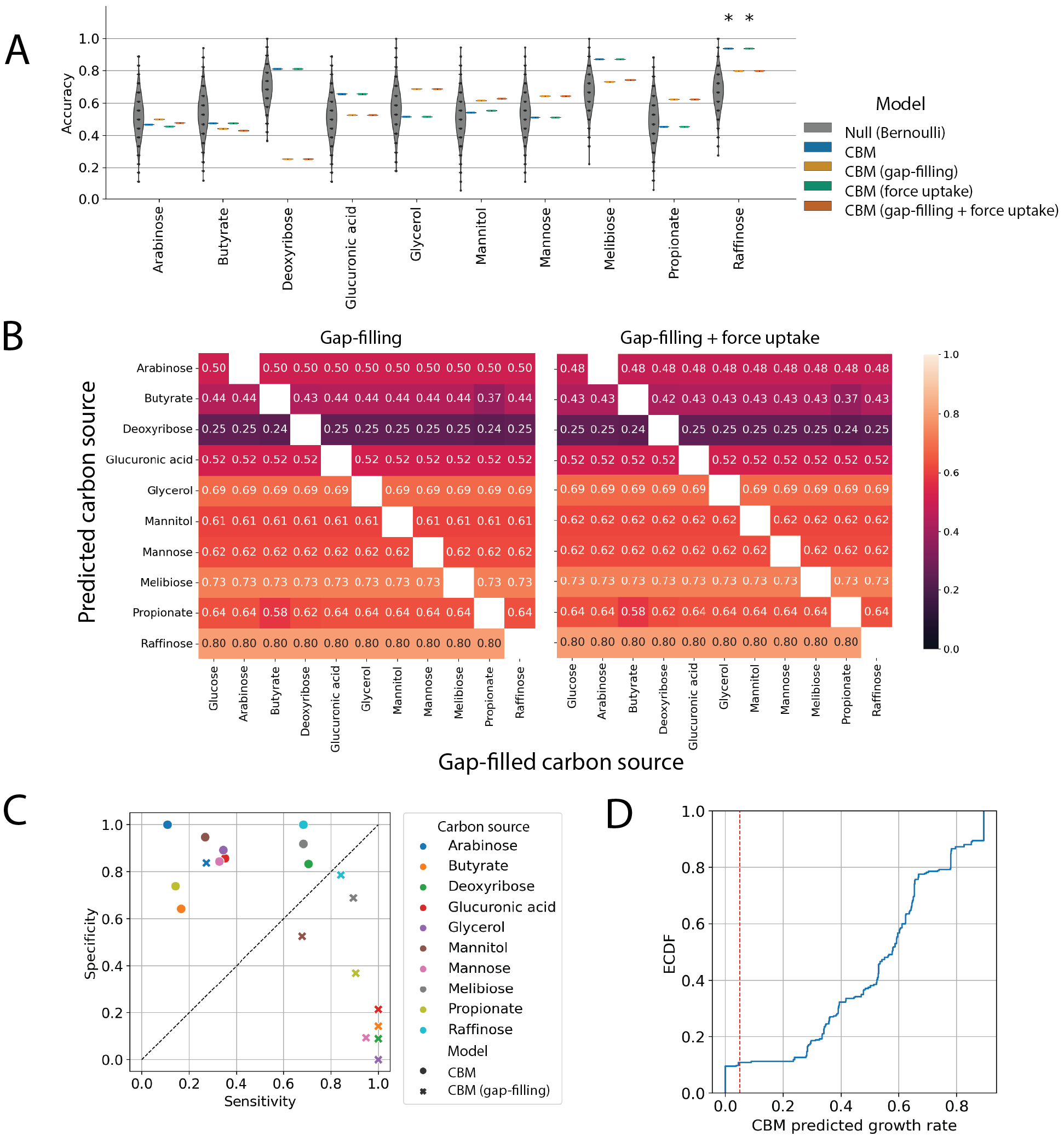

Supplement: S2 Fig — (A) Gap-filling or forcing uptake did not improve CBM prediction accuracy. Grey violin plots: distribution of accuracy of the Bernoulli null model. CBM predictions with significantly higher accuracy than the null model after multiple-testing correction (Methods) are marked *. (B) The choice of carbon source used for gap-filling did not significantly alter the prediction outcome. The left panel shows the prediction accuracy of CBM models gap-filled with one carbon source (x-axis) on the growth with another carbon source (y-axis). The right panel shows the same results while forcing uptake reactions for the predicted carbon source from the medium to the cytoplasm. Entries with the same gap-filling carbon and prediction carbon are omitted because these metabolic models always predict positive growth. (C) Sensitivity and specificity of CBM models. While gap-filling or not yielded similar prediction accuracy, non-gap-filled CBM models had a high false negative rate (low sensitivity) while gap-filled CBM models had a high false positive rate (low specificity). (D) Distribution of CBM predicted growth, defined as the flux through the biomass reaction. Red line: our choice of growth threshold (0.05 mmol per gram dry weight of cells and hour). Changing the growth threshold did not significantly affect the prediction results. (TIF) [file pcbi.1011705.s002.tif]

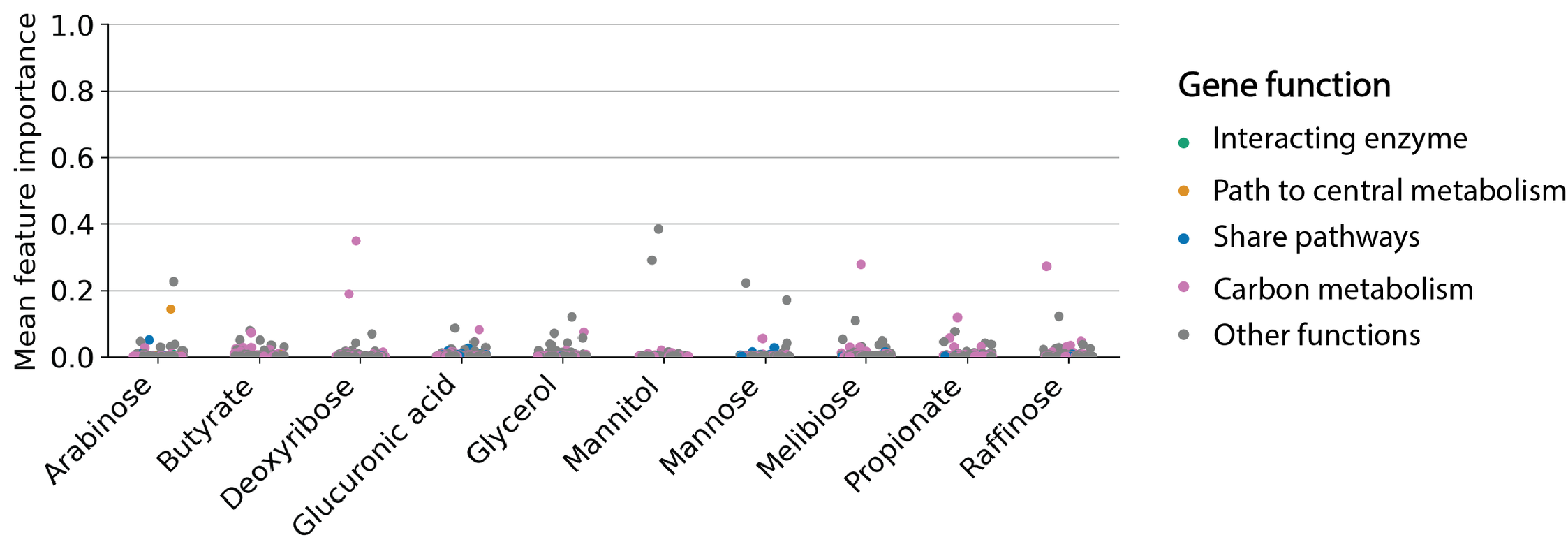

Supplement: S3 Fig — Gene feature importance scores for the 10 carbon sources in random forests trained under random data partitions (blue in Fig 2B). For each gene, the feature importance score in the y-axis was calculated as the mean feature importance score of 100 random forests trained in the 100 random data partitions. Gene functions are highlighted by color and were assigned using KEGG pathways (see Methods). Note that although random forests show high prediction accuracy in Fig 2B, these predictions often did not rely on enzymes related to the metabolism of the target carbon source. (TIF) [file pcbi.1011705.s003.tif]

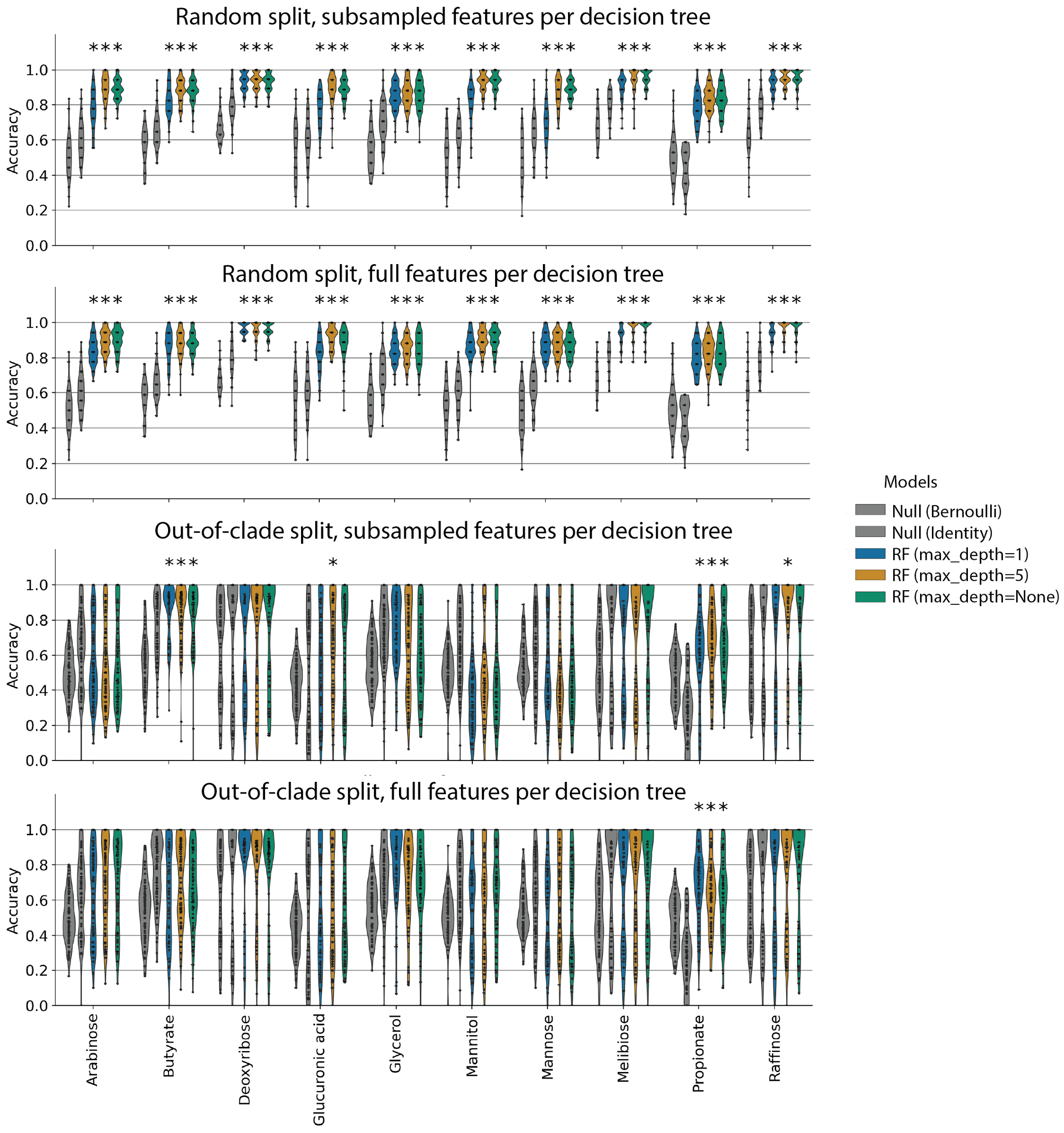

Supplement: S4 Fig — We tuned two key hyperparameters in random forests models: maximum tree depth and feature number used per decision tree (Methods). Varying these hyperparameters did not improve random forest predictions in both randomly held out test sets (top) and out-of-clade test sets (bottom). (TIF) [file pcbi.1011705.s004.tif]

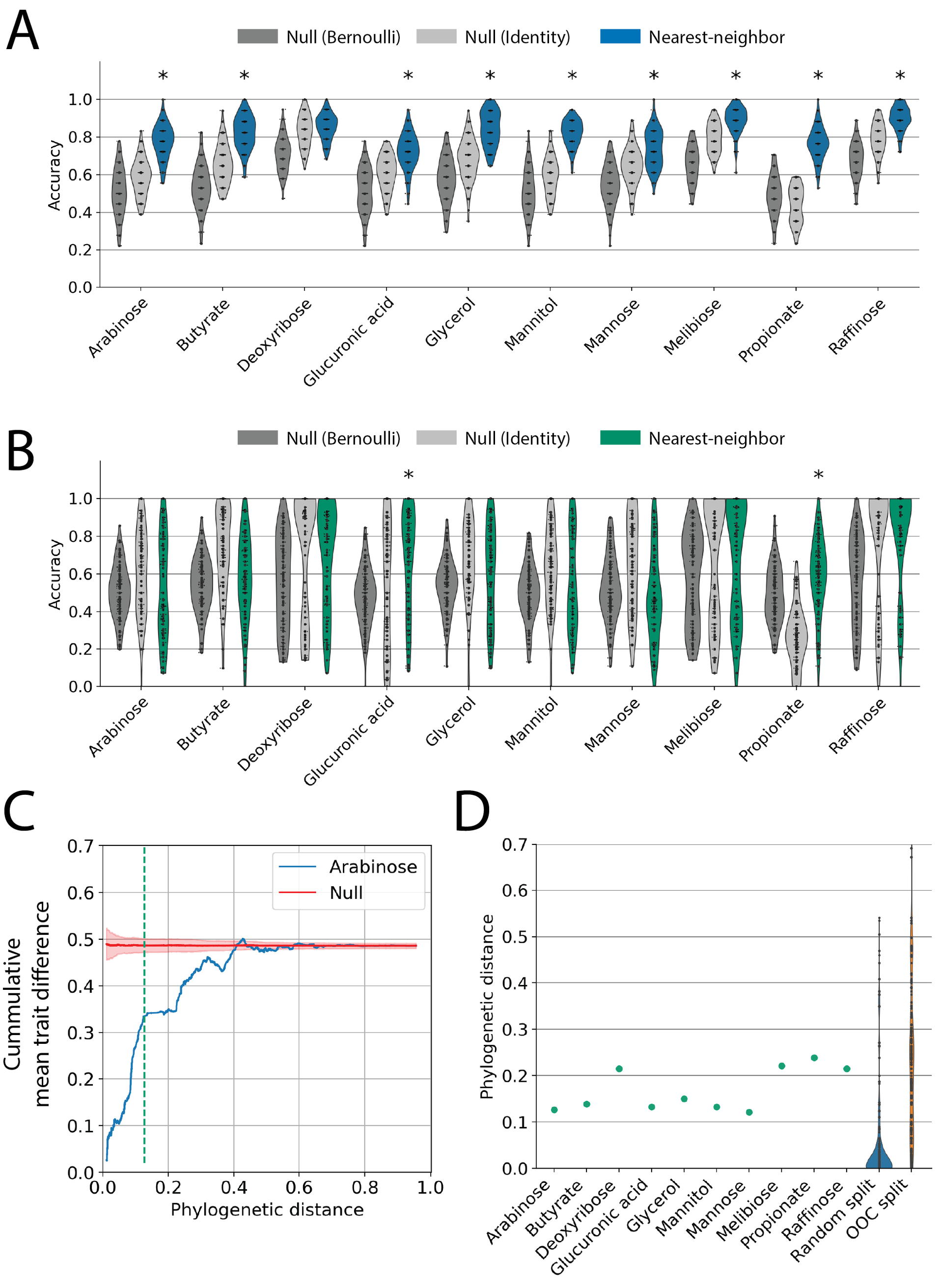

Supplement: S5 Fig — (A) Trait prediction accuracies by nearest neighbor classifiers using 16S rRNA distances (hamming distance; see Methods) as predictors (blue) with randomly partitioned training/test sets as in Fig 2A. Predictions are tested against two null models (Bernoulli, identity; see Methods), and those that significantly outperform both null models are marked * (p < 0.05 after a multiple-testing correction; see Methods). (B) Trait prediction accuracy for out-of-clade test sets (as in Fig 2C), using nearest neighbor classifications on 16S rRNA sequence (green). Predictions that significantly outperform both null models are marked * (p < 0.05). (C) Phylogenetic correlation of the arabinose utilization trait. X-axis is the phylogenetic distance between strains. Y-axis is the average difference in trait below the distance, calculated as follows: at a given phylogenetic distance, we identify all strain pairs with distances below the value and calculate their mean difference of traits (1 for different traits and 0 for identical trait—growth/no-growth). The blue curve shows the mean trait difference as a function of the phylogenetic distance in our data. The red curve shows the expected curve of a null model with randomly distributed traits and the red-shaded region indicates the standard deviation over 100 bootstrapping instances. The green dashed line indicates the correlation length scale for arabinose calculated using chi-square statistics (Methods). (D) The correlation length scale for the 10 carbon sources (green markers). Violin plots show the distribution of phylogenetic distances between test strains and their training nearest neighbors for random partitions of the data (blue) and out-of-clade partitions (orange). (TIF) [file pcbi.1011705.s005.tif]

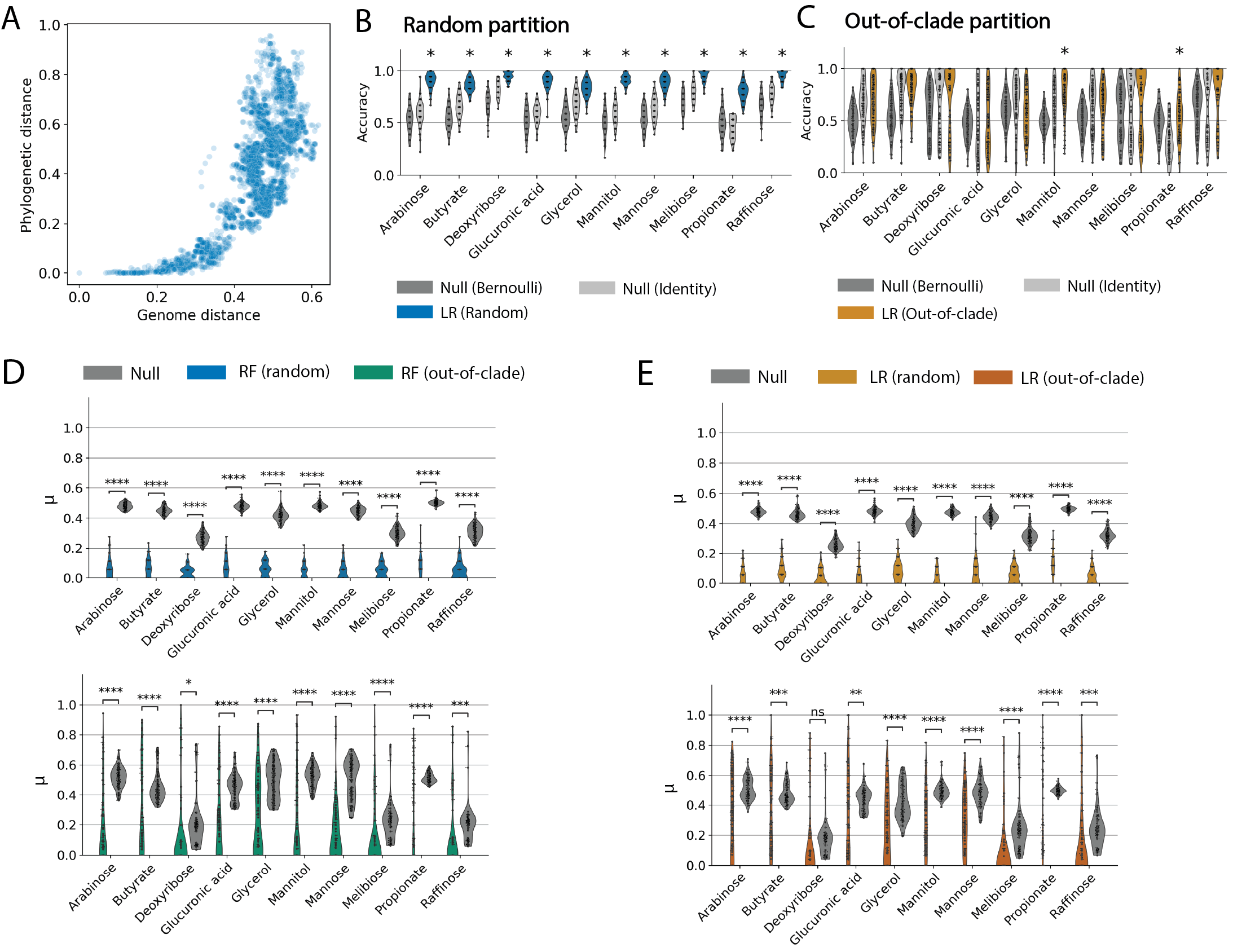

Supplement: S6 Fig — (A) The genome similarity, defined by the L1 distance of gene presence-absence vectors for a pair of strains (x-axis), and the phylogenetic distance, defined by the hamming distance between 16S rRNA sequence for a pair of strains (y-axis), for all strain pairs. The genome distance strongly correlates with the phylogenetic distance. Therefore, nearest-neighbor models using gene presences-absence performed similarly to those using 16S rRNA sequences (compare Figs 2B, 2C and S5(A) and S5(B)). (B)(C) Logistic regression, like the random forest, predicts traits accurately under random data partitions (B) but not for out-of-clade partitions (C). (D) Comparing random forests’ prediction on test samples and their training nearest neighbors. We compute the mean predicted trait difference between test samples and their L1 training nearest-neighbors, on both random partition (green, left panel) and out-of-clade partition (blue, right panel, see Methods for details). Compared to a null model (grey) of picking a random strain in the training set instead of the nearest-neighbor by genome similarity (L1 distance of gene presence-absence vectors), RF tends to make the same prediction for a test sample and the training sample with the most similar genome, consequently behaving like a nearest-neighbor model using L1 genome distances. Bars on the top indicate p-values, computed by permutation t-tests (two-sided, 105 permutations) and corrected for multiple testing on 10 carbon sources (Methods). (E) Similar to random forests in (D), logistic regression also tends to make the same predictions for test samples and their training nearest neighbors, for both random (light orange, left panel) and out-of-clade partitions (dark orange, right panel). see Methods for details. (TIF) [file pcbi.1011705.s006.tif]

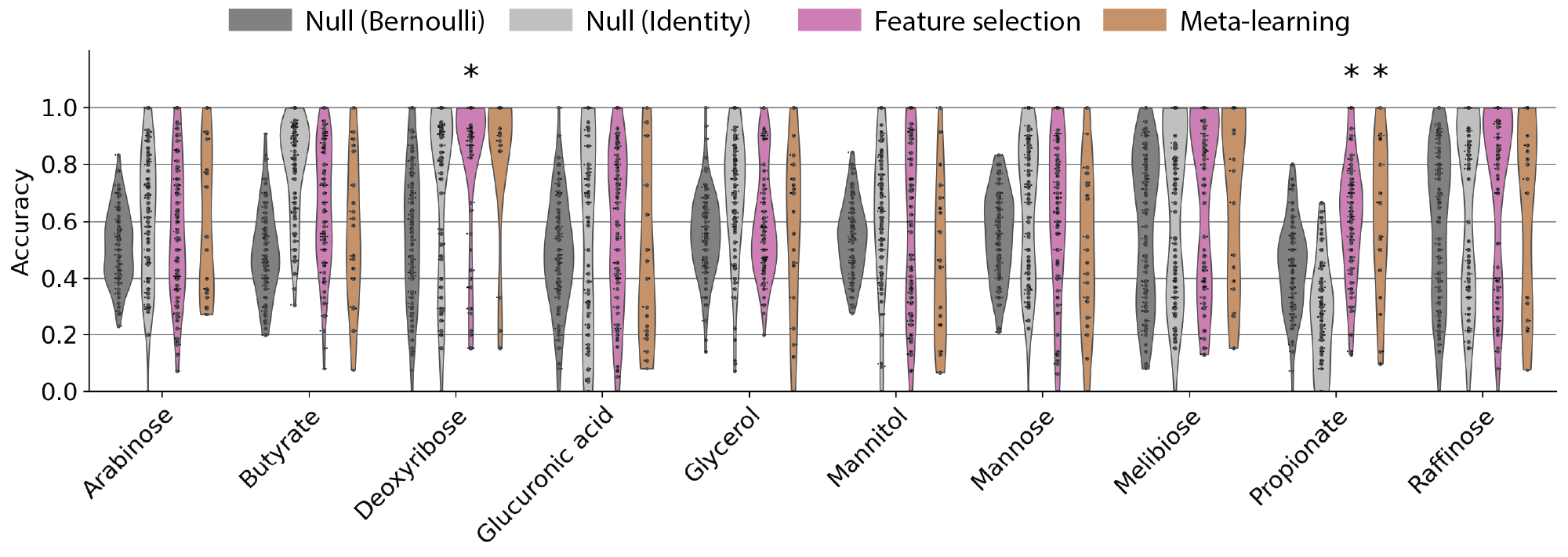

Supplement: S7 Fig — The greedy feature selection method (pink; Methods) and the meta-learning approach (orange; Methods) were tested on out-of-clade test sets. Each model is cross-validated over 100 data partitions and plots show the distribution of prediction accuracy. Predictions that significantly outperform both null models at the same data partition are marked * at the top (p < 0.05 after multiple-testing corrections; see Methods.) (TIF) [file pcbi.1011705.s007.tif]

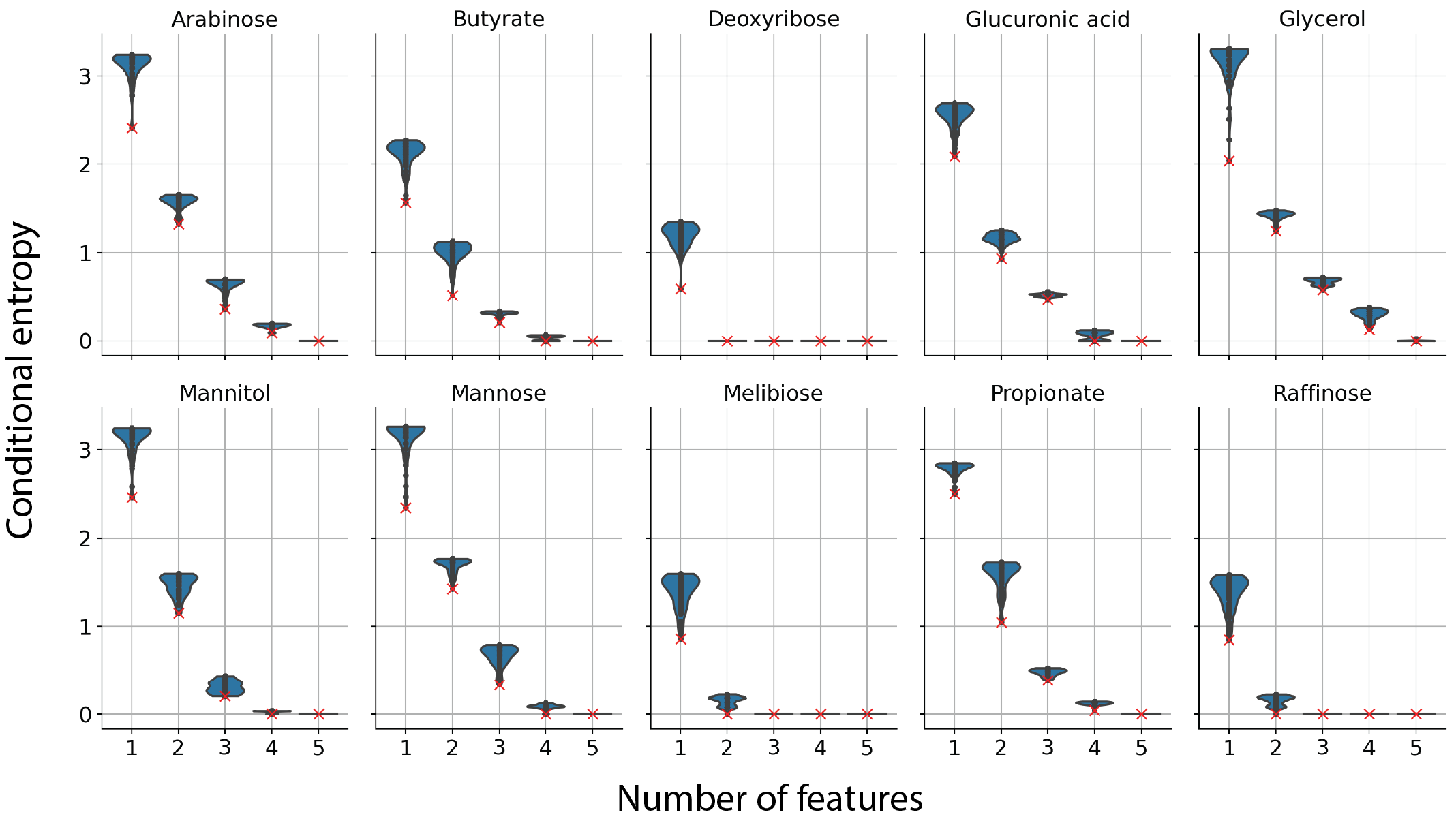

Supplement: S8 Fig — Violin plots show the distribution of conditional entropy (Methods) between the top 100 combinations of one to five genes with the lowest conditional entropy. The combination with the lowest conditional entropy is marked as the red cross. Note that as the number of genes increases, the vertical extent of the violin plot decreases, indicating the top 100 combinations all have similar conditional entropy with the trait. This means that when 5 genes are selected, all 100 choices of the 5 genes are equally predictive of the trait. In contrast, for only one gene the best predictor (red “x”) is unique in that it is an outlier in the distribution, but this gene presence/absence has high conditional entropy with the target trait and is therefore a poor overall predictor. We argue that this redundancy effect, as a result of the small number of samples and the large number of predictors, prevents effective feature selection. (TIF) [file pcbi.1011705.s008.tif]

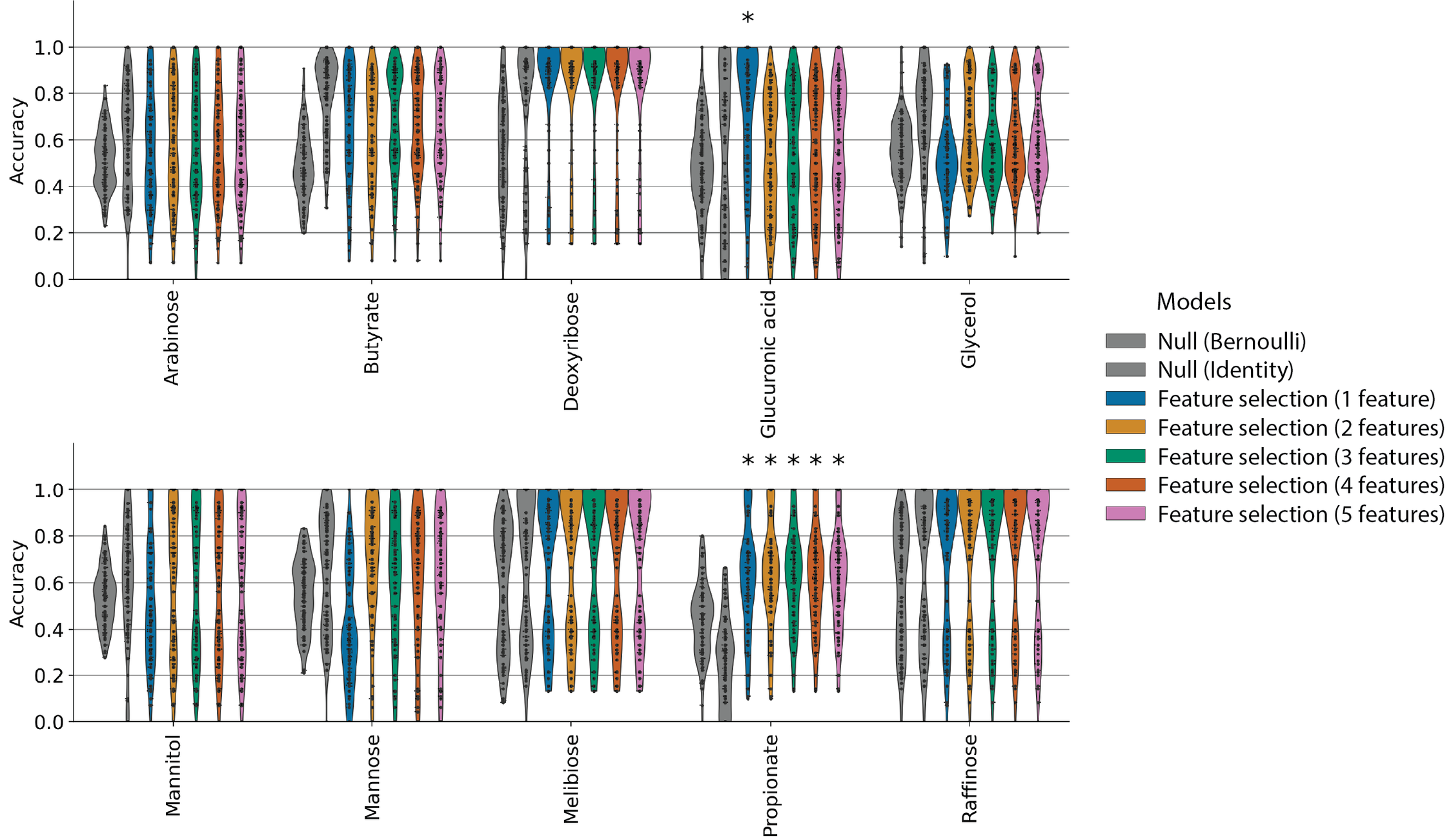

Supplement: S9 Fig — Prediction accuracy of the random forest classification with different numbers of selected genes, under out-of-clade partitions with different numbers of features. Changing the number of selected features did not improve the model’s performance. (TIF) [file pcbi.1011705.s009.tif]

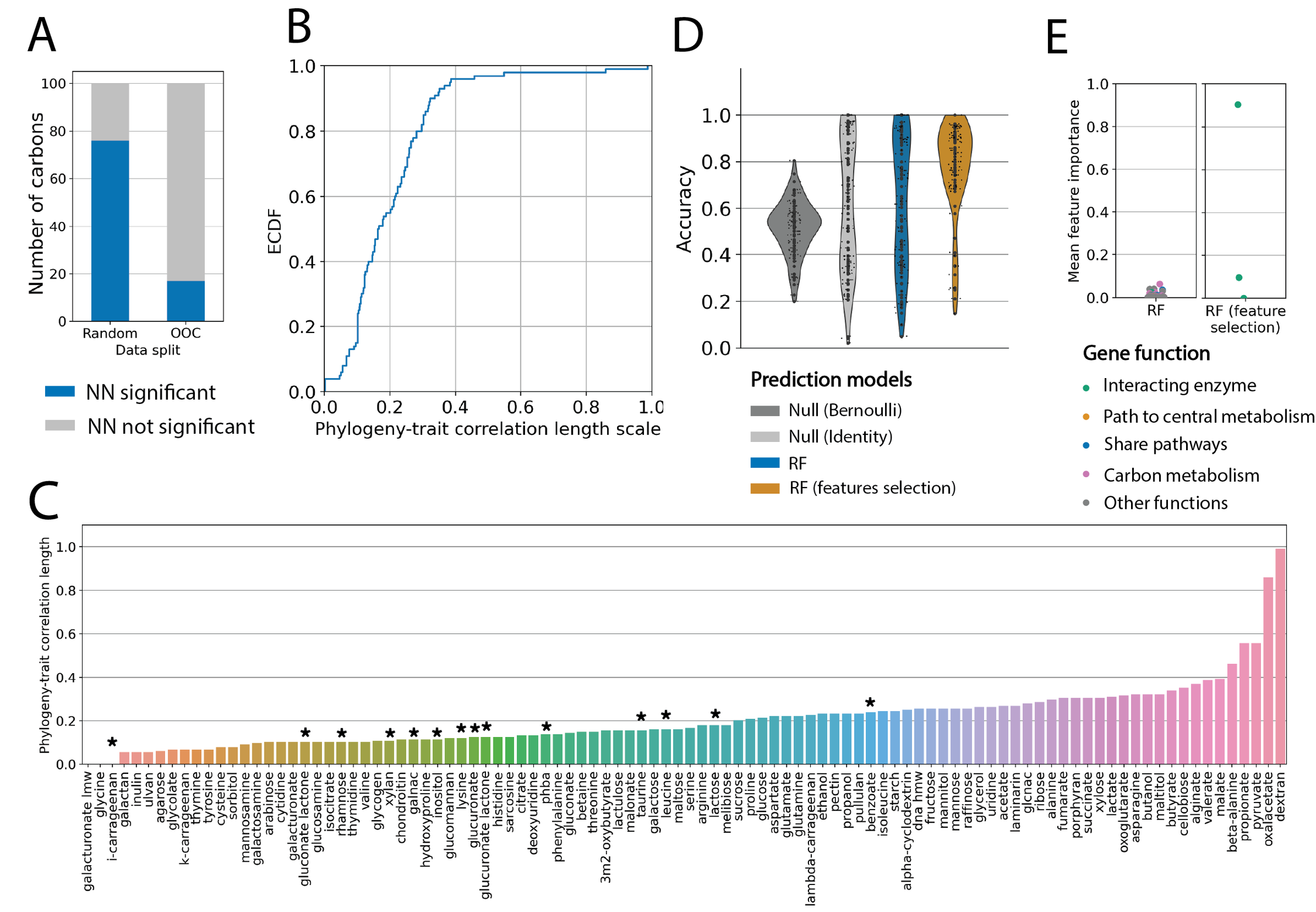

Supplement: S10 Fig — (A) Using 16S sequences alone as predictors, the phylogenetic nearest-neighbor classifier successfully predicted growth on 76 out of 100 carbon sources when the dataset is partitioned randomly yet only 24 out of 100 carbon sources when the dataset is partitioned out-of-clade. A prediction is considered successful when the accuracy over 100 data partitions is significantly higher than the two null models by a permutation t-test (105 permutations) after multiple testing corrections for the 100 carbon sources (p < 0.05; see Methods). (B)(C) The empirical cumulative distribution function (ECDF) of the phylogeny-trait correlation length scale calculated for the 100 carbon sources (see S5(D) Fig and Methods). If we fail to detect correlation, the length scale value is assigned 0. (C) list the phylogeny-trait correlation length scales for all 100 carbon sources. The 14 carbon sources where RF outperformed KO nearest-neighbor models in out-of-clade prediction (Fig 3C) are highlighted with *. Their correlation length scales are shorter than the other carbon sources (p = 0.03, permutation t-test). (D) Comparison of random forest out-of-clade prediction on isoleucine utilization using gene presence-absence, without (blue) or with feature selection using KEGG (orange). While both predictions are significant compared to the two null models (grey), the feature selection using KEGG by restricting predictors to only genes interacting with the carbon compound significantly improved the prediction accuracy (compare orange to blue; p = 0.0036, permutation t-test with 105 permutations after multiple-testing correction.) (E) Feature importance scores for random forests trained in panel (D) without (left) or with (right) feature selection. Features selection forced the random forest to use key metabolic enzymes instead of using phylogenetic signals (note the gene with a high feature importance score is an enzyme that interacts with isoleucine), resulting in improved prediction accuracy. [file pcbi.1011705.s010.tif]

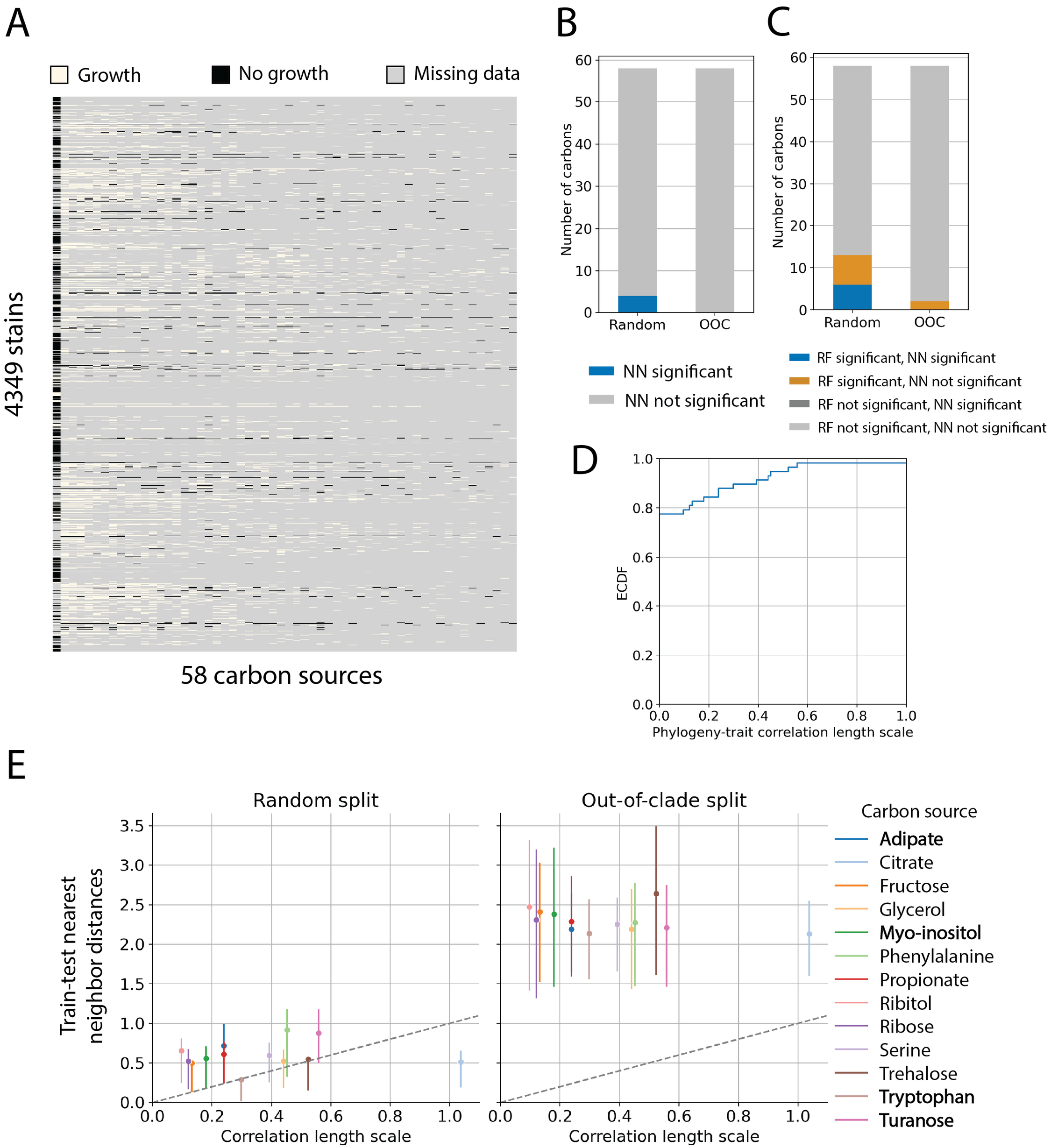

Supplement: S11 Fig — (A) The curated dataset consists of binary utilization data spread across 4349 diverse microbes on 58 carbon sources, with sample size for each carbon source ranging from 104 to 2394 samples. The matrix shows growth/no-growth data as in Fig 1. (B) Using 16S sequence distances to predict traits. The phylogenetic nearest-neighbor model using only 16S sequences as predictors outperformed both null models (Bernoulli and identity) on only 4 out of the 58 carbon sources for the random partition and none for the out-of-clade partition. (C) Use KO presence-absence to predict traits. When the training sets and test sets are partitioned randomly, random forest outperformed both null models (Bernoulli and identity) on 13 (blue and orange) out of the 58 carbon sources and 6 carbon sources (blue) were also significantly predicted by nearest neighbor classifiers. When the dataset is partitioned out-of-clade, only 2 carbon sources were significantly predicted by random forest (orange) and none was significantly predicted by nearest-neighbor. (D) Empirical cumulative distribution function (ECDF) of the phylogeny-trait correlation length scale for the 58 carbon sources. We failed to detect correlation for 45 out of the 58 carbon sources, for these carbon sources we assigned a value of 0. (E) Comparing train-test nearest-neighbor distances with the phylogeny-trait correlation length scale for the 13 carbon sources with measurable phylogeny-trait correlation. Similar to S5(D) Fig, for each carbon source, we divided the available data either randomly or out-of-clade and, for each sample in the test set, found its phylogenetically closest neighbor in the training set and calculated their phylogenetic distance (the branch distances between the two nodes on the tree). For each carbon source, we compared the distribution of the train-test nearest neighbor distance (y-axis; point shows the mean and the error bar shows the 50% percentile interval) with the phylogeny-trait correlation length [file pcbi.1011705.s011.tif]

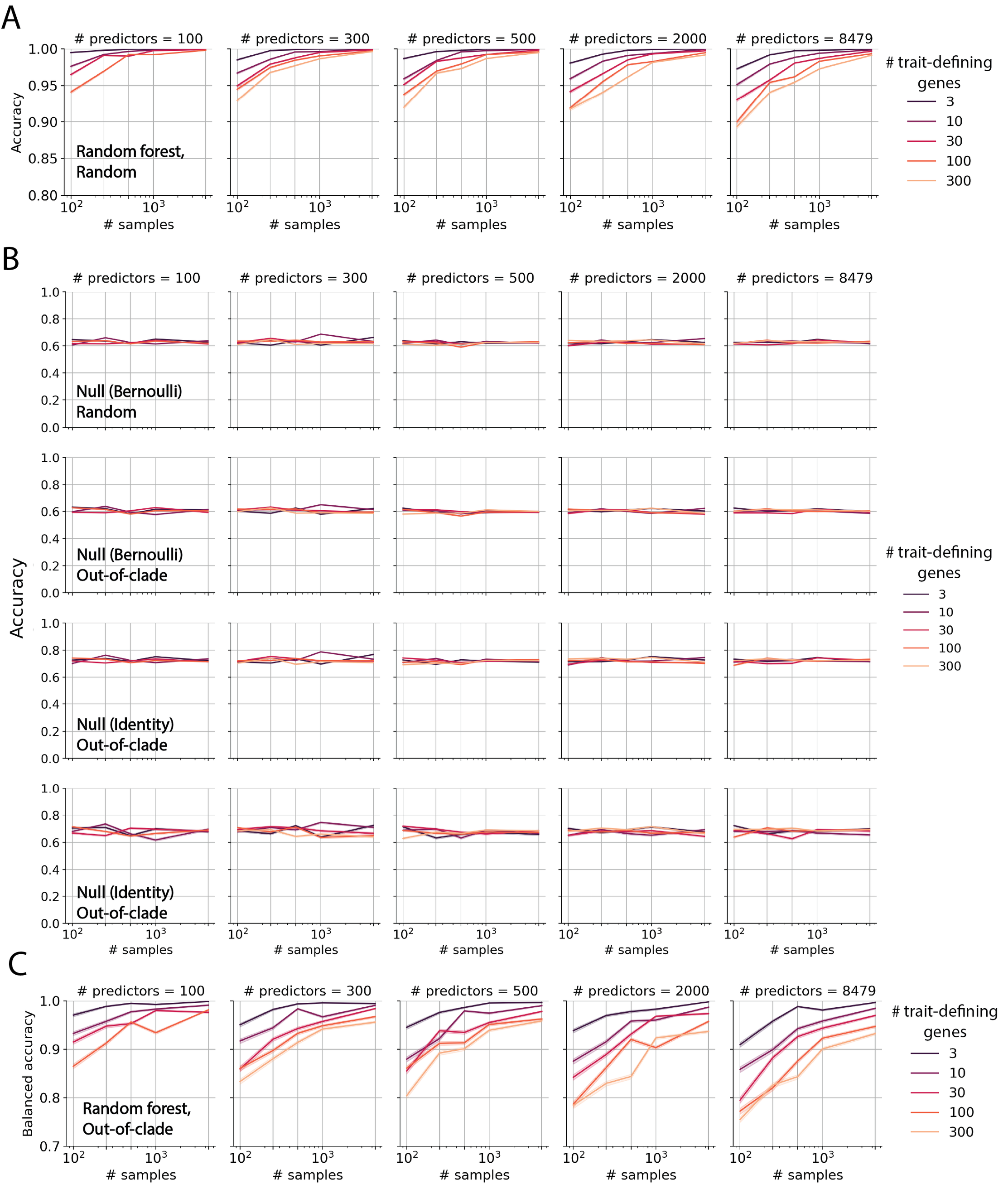

Supplement: S12 Fig — (A) Random forest prediction accuracy for synthetic traits under random partitions with synthetic datasets with different properties, including different sample sizes (x-axis), different numbers of predictors (different panels), and different trait complexities (different colors). Note the higher prediction accuracy compare to the out-of-clade prediction accuracy in Fig 5E. (B) Prediction accuracy of the Bernoulli null model and the identity null model under random and out-of-clade partitions. Note the lower prediction accuracy compare to panel (A) and Fig 5E. (C) Balanced accuracy, defined as the arithmetic mean of sensitivity and specificity, of random forest predictions on out-of-clade partitions (same scenario as Fig 5E). Note the high balanced accuracy score, which indicates that the high accuracy score in Fig 5E is not an effect of unbalanced fractions of zeros and ones in the trait data. (TIF) [file pcbi.1011705.s012.tif]

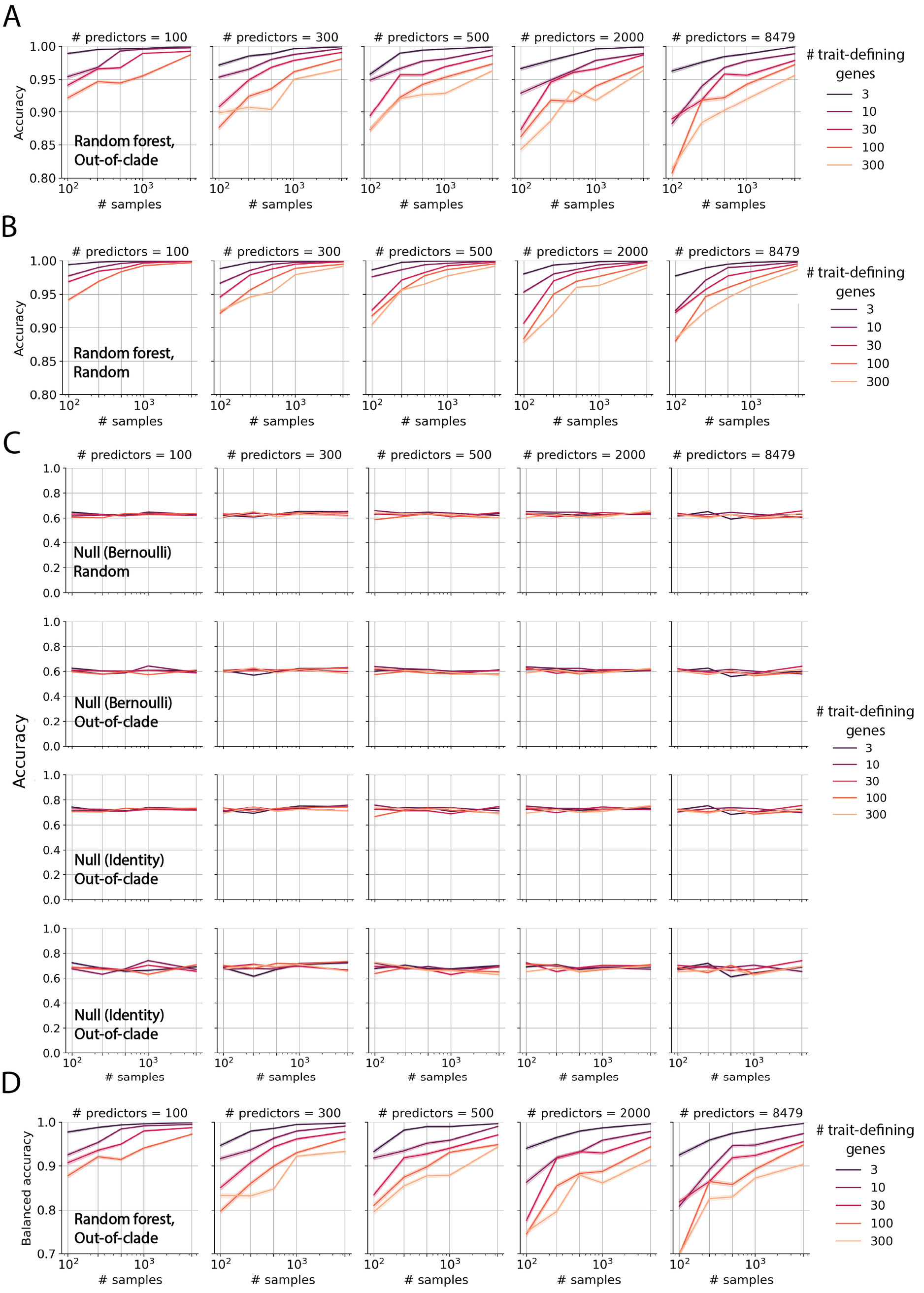

Supplement: S13 Fig — The model prediction accuracy on synthetic data with traits at modularity 0.3 (see Methods and the supplementary code for details). Note that for synthetic datasets with the same sample size, the same number of predictors, and the same number of trait-defining genes, predicting modular traits (this figure) has a lower prediction accuracy than predicting non-modular traits (modularity = 0; Figs 5E and S12). Each panel shows similar information as Figs 5E and S12: (A) Random forest prediction accuracy for synthetic datasets under out-of-clade partitions. (B) Random forest prediction accuracy for various synthetic datasets under random partitions. (C) Bernoulli null and identity null prediction accuracy on random or out-of-clade partitions. (D) Balanced accuracy of random forest prediction on out-of-clade partitions. (TIF) [file pcbi.1011705.s013.tif]

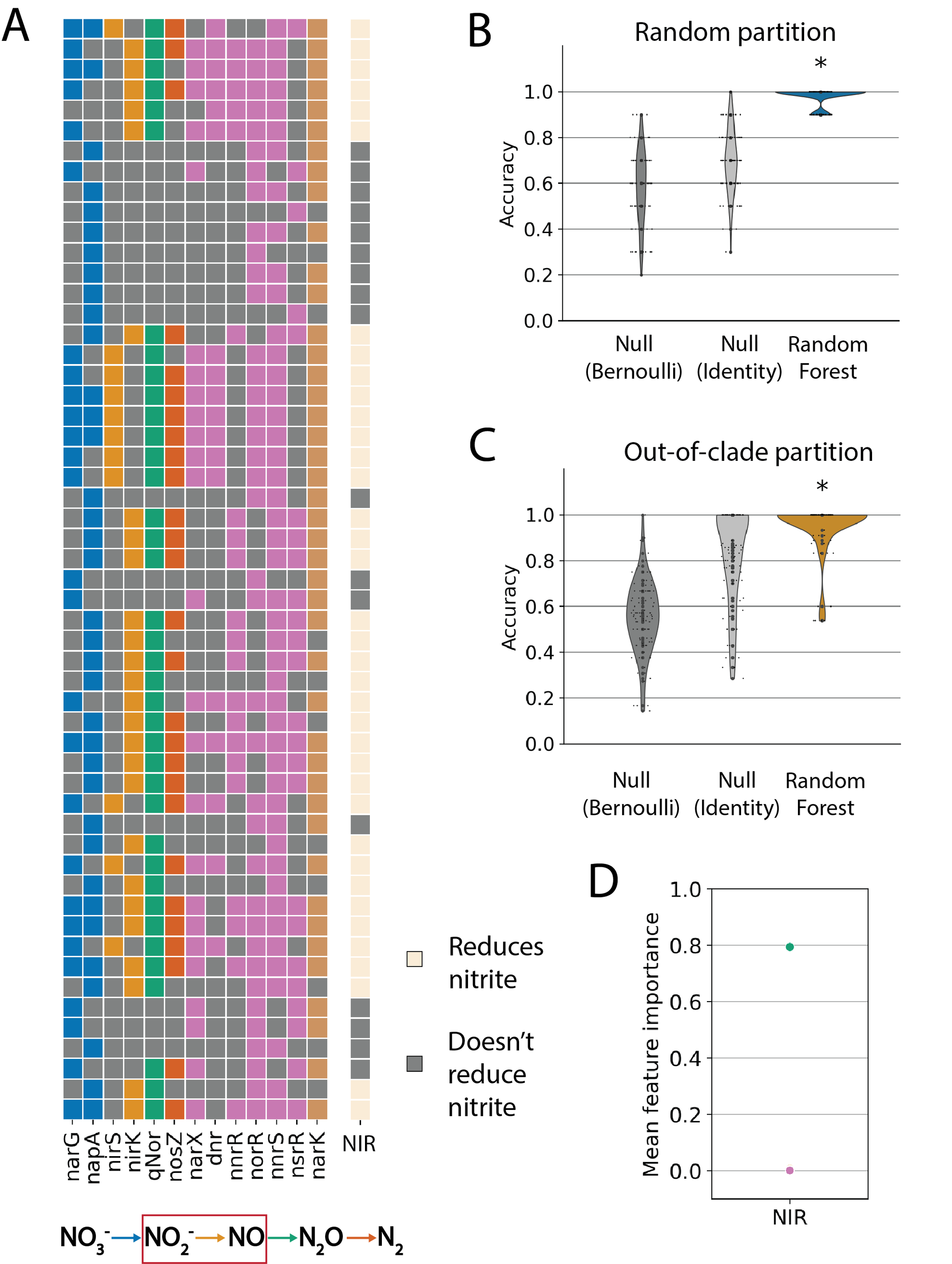

Supplement: S14 Fig — (A) Presence-absence of denitrification-related genes of 54 strains (left matrix) and their nitrite reduction capability (right vector) from Gowda et al. [19]. Blue: nitrate reductases; orange: nitrite reductases; green: nitric oxide reductase; red: nitrous oxide reductase; pink: regulators; brown: transporter. Gene colors correspond to different reactions in the denitrification cascade illustrated at the bottom. Note that the gene vector (nirS or nirK) is almost identical to the qNor gene vector, which is almost identical to the nitrite reduction trait (NIR, last column). (B)(C) Prediction of whether strains grow on nitrite (via anaerobic respiration) from the full genomes using nearest-neighbor models and random forest models for both random (B) and out-of-clade partitions (C). (D) Mean feature importance of genes in the random forest models trained out-of-clade partitions. The nitric oxide reductase, qNor, is used as the strongest predictor in random forests. (TIF) [file pcbi.1011705.s014.tif]
