# Supplementary material for: Statistical prediction of microbial metabolic traits from genomes
Source: PLoS Comput Biol. 2023 Dec 19;19(12):e1011705. doi: 10.1371/journal.pcbi.1011705 (PMC10729968; doi:10.1371/journal.pcbi.1011705)
Supplement: S1 Table — (PDF) [file pcbi.1011705.s015.pdf]

S1 Table. Summary of prediction results.

Dataset	Method	Dataset partition	Number of carbon sources with significant prediction	Notes
This study	Constraint-based modeling	NA	0/10	
This study	16S nearest-neighbor	Random	9/10	
This study	16S nearest-neighbor	Out-of-clade	2/10	
This study	KO nearest-neighbor	Random	10/10	
This study	KO nearest-neighbor	Out-of-clade	1/10	
This study	Random forest	Random	10/10	
This study	Random forest	Out-of-clade	2/10	
This study	RF + KEGG feature selection	Out-of-clade	8/10	
Gralka et al.	16S nearest-neighbor	Random	76/100	
Gralka et al.	16S nearest-neighbor	Out-of-clade	13/100	
Gralka et al.	KO nearest-neighbor	Random	84 / 100	
Gralka et al.	KO nearest-neighbor	Out-of-clade	24/100	
Gralka et al.	Random forest	Random	88/100	
Gralka et al.	Random forest	Out-of-clade	36/100	RF outperformed KO NN on 14 carbon sources.
Gralka et al.	RF + KEGG feature selection	Out-of-clade	43/100	Improved out-of-clade prediction for 11 carbon sources compared to RF.
BacDive	16S nearest-neighbor	Random	4/58	
BacDive	16S nearest-neighbor	Out-of-clade	0/58	
BacDive	KO nearest-neighbor	Random	6/58	
BacDive	KO nearest-neighbor	Out-of-clade	0/58	
BacDive	Random forest	Random	13/10	
BacDive	Random forest	Out-of-clade	2/10	
