# Supplementary material for: Statistical prediction of microbial metabolic traits from genomes
Source: PLoS Comput Biol. 2023 Dec 19;19(12):e1011705. doi: 10.1371/journal.pcbi.1011705 (PMC10729968; doi:10.1371/journal.pcbi.1011705)
Supplement: S2 Table — (PDF) [file pcbi.1011705.s016.pdf]

S2 Table. Modified 1/5x TSB medium composition

Compound	Concentration
Tryptone	3.4g/L
Soytone	0.6g/L
NaCl	17.11mM
K_2HPO_4	2.871mM
Glucose	2.775mM
