# Supplementary material for: Statistical prediction of microbial metabolic traits from genomes
Source: PLoS Comput Biol. 2023 Dec 19;19(12):e1011705. doi: 10.1371/journal.pcbi.1011705 (PMC10729968; doi:10.1371/journal.pcbi.1011705)
Supplement: S3 Table — (PDF) [file pcbi.1011705.s017.pdf]

S3 Table. C-free medium composition

Compound	Concentration
Na_2HPO_4	28.61mM
KH_2PO_4	11.39mM
$(NH_4)_2SO_4$	7.568mM
Nitrilotriacetic acid	1.046mM
$MgSO_4$	2.401mM
$CaCl_2$	0.4537mM
$(NH_4)_6Mo_7O_2$	0.1497μM
$FeSO_4$	7.122μM
$Na_2 \cdot EDTA$	8.554μM
$ZnSO_4$	38.25μM
$FeSO_4$	17.98μM
$MnSO_4 \cdot H_2O$	9.111μM
$CuSO_4$	1.574μM
$CoCl_2$	0.8490μM
$Na_2B_4O_7$	0.4641μM
Yeast Extract	0.05g/L
