# Supplementary material for: Statistical prediction of microbial metabolic traits from genomes
Source: PLoS Comput Biol. 2023 Dec 19;19(12):e1011705. doi: 10.1371/journal.pcbi.1011705 (PMC10729968; doi:10.1371/journal.pcbi.1011705)
Supplement: Attachment — Submitted filename: PLoS_Comp_Bio_ReviewResponse.pdf [file pcbi.1011705.s018.pdf]

October 6th, 2023

Dear Christos and Zhaolei,

Thank you for taking the time to review our manuscript. We have addressed the comments from the reviewers and we hope you find the resulting manuscript suitable for publication. Below we detail a point by point response to the questions posed by the referees. As requested, we are submitting a revised manuscript with revisions in red, and a clean version of the paper without revisions demarcated.

Regards,

Zeqian Li, Ahmed Selim, Seppe Kuehn.

We made the following changes in addition to the reviewer's suggestion:

- Updated the reference to Gralka et al. The paper was published in Nature Microbiology recently. (This change is not highlighted in red due to latex formatting difficulty).
- Updated funding information (line 540)

Comments to the Authors:
Please note here if the review is uploaded as an attachment.

Reviewer #1: This manuscript by Li et al discusses various methods to predict metabolic function using genomes. The main findings are that phylogenetic predictions are accurate provided that you have a close match in your database and that very large datasets can enable stronger, more mechanistic, and phylogeny-independent predictions. The writing is clear and easy to follow, and the conclusions are generally well supported by the data. The paper is timely, as available phenotypic and genomic data sets are poised to grow, increasing the usability of machine learning methods for phenotypic predictions.

The novelty of this work is murky, as the main findings are generally well known to science. The phylogenetic conservation of metabolism is well known; clinical microbiologists have been using metabolism to taxonomically label organisms for many decades. It is also generally well appreciated that larger data sets are required for accurate machine feature lists are long, and that without sufficiently large data overfitting (e.g. learning the wrong genes) can be a problem. Regardless, the unifying framework in which these lessons are discussed and the clarity with which they are discussed may prove of value to the large group of researchers getting started on machine learning or microbial predictions from genomes.

Suggestions for improvement:

- More discussion of the well-known relationship between phylogeny and metabolism (e.g. use in the clinic) would be appreciated in the results and/or discussion. Right now it is primarily discussed as why out-of-clade fails – as almost a negative prior on phylogeny being informative, overplaying the novelty of this work.

    - We added discussion in the main text (line 233) and discussion (line 482) to address this comment.

- The authors note 14 carbon sources for which the RF classifier does well out-of-clade when using gene/presence absence but for which a nearest neighbor approach on the same underlying data does not do well. Are these 14 carbons sources the most likely to vary *within clades*, forcing the RF model to learn something independent of phylogeny? This might help us understand the kinds of datasets that are needed—for example, data sets that include deep sampling within clades might be particularly useful.
    - This is a great point. In the original text, we argued that the 14 carbon sources have better annotated metabolic pathways. However following the reviewer's suggestion, we looked at how traits vary within the phylogenetic clades and found that the 14 carbon sources have a weaker trait-phylogeny correlation compared to the other carbon sources. As a result, they have more trait variation during model training. We hypothesize that both factors contributed to their success. We added a discussion of this topic at line 257 and modified figure S10 (and caption).
- A graph that compares all various discussed methods side by side might be particularly useful. It is hard to graphically understand how various methods compare to one another.
    - A summary table was added the compare all datasets and methods (page 73; referenced at line 490)
- Gralka et al report more strains and carbon sources than listed in this paper. Is there a reason for this?
    - This was discussed originally in the method section. We added a clarification in the main text (line 207)
- Page 11: It seems that the KO presence absence is actually slightly better than 16S alone (84 vs 76; 24 vs 13)? Why do the authors downplay this?
    - This is a great point. We hypothesize that this is because similarity in KO presence-absence capture more information besides phylogeny, such as sugar-acid preference detailed in Gralka et al., which is helpful in predicting traits. Added discussion at line 224.
- Page 8, top: After discussion of very well thought out metrics, it is not discussed how the just discussed classifier does on those metrics (Its in the figure but missing from the text).
    - We have added a discussion of this point at line 156.
- Page 8, bottom: Figure 5 referenced, I believe this is Figure S5.
    - Fixed (line 185)
- Page 11: For the sentence beginning with "Training nearest-neighbor classifiers using KO…" is not clear what the numbers in parentheses represent as comparators too, as no small numbers akin to 24/100 are presented earlier in the paragraph.
    - Fixed (line 221)
- Page 18, second paragraph: predicts->predict
    - Fixed (line 352)
- Page 19: It is not clear to me if rnfG is being used to predict phylogeny, perhaps I am missing something.
    - We understand that this is confusing because we mentioned that rnfG improved prediction in certain orders. Our argument is the following: rnfG is *not* merely used as a phylogeny marker, because it the successful in predicting out-of-clade. We argue

that the model trained including rnfG learned a generalizable mechanism, and the mechanism may only be important to tryptophan utilization in certain clades. A brief discussion was added at line 388.

- Page 20: Top paragraph, extra “used” in “studied used”
  - Fixed (line 443)
- Page 24: The sentence “In particular, we show..” should have a qualification that says “some carbon sources”
  - Fixed (line 475)
- Page 24: Not clear for a naiive reader what “respect the phylogenetic structure” means, perhaps a better verb would be more clear.
  - Clarified the statement (line 495)

Reviewer #2: Finding genomic signatures of microbial metabolic activity from communities is extremely challenging and directly linking microbial traits to environment and host health is the holy grail of microbial research. This paper successfully attempts to predict microbial traits from their genomic content from culture isolates and existing sequencing datasets – a clever combination of approaches with novel statistical tools.

The authors report a combinations of study approaches that go from simple to complex, leveraging robust statistical tools. They also use multiple datasets to test and verify the predictions. While I am not an expert in the mathematical aspects of the paper, I can vouch for the microbial mechanistic underpinnings of the approach. The paper uses strong theory and is based on clear microbial metabolic mechanisms. The focus on fundamental metabolic pathways makes it highly applicable to different sub-disciplines of microbiology.

The paper investigates the statistical links of genes to traits, sometimes using phylogenetic information to make those linkages, but what is more powerful is the attempt to look for the underlying mechanisms that examines the reason behind that statistical linkage. For example, the exploring of carbon utilization gene presence/absence patterns in the marine dataset to predict traits and how phylogenetic information is exploited to make predictions. The authors also present multiple examples of specific substrates or phylogenetic subsets to explain the mechanistic underpinning of their statistical predictions which makes the paper easy to read and understand. I also particularly liked the use of denitrification to test the random forest model and authors suggestion that the model does not necessarily detect mechanistically linked genes directly responsible for the trait but rather other genes that many be correlated with it. Indeed, in some cases the linkages are not linked to exact genes responsible for the traits and the mechanism of interest. But as the authors themselves suggest sometimes its ok to simply detect a gene that can be used to predict a process without knowing the underlying mechanism of the link of the gene to the process.

Authors discuss the results very well and provide some suggestions and recommendations for future work. This work will certainly be useful in guiding experiments and modelling endeavours to upscale from single cells to complex communities.

Methods are provided in detail, the data used in the paper are publicly available so is the data analysis code. I haven’t explored this in detail but skimming through I get the impression that it is well structured and easy to access.

It is quite a rare case that I am not making any suggestions to improve the paper. It is quite a polished manuscript ready for publication.

- This is the first time the PI has submitted any manuscript that received this feedback. We are thankful for the careful review and the positive comments on the manuscript.
