# Supplementary material for: Statistical prediction of microbial metabolic traits from genomes
Source: PLoS Comput Biol. 2023 Dec 19;19(12):e1011705. doi: 10.1371/journal.pcbi.1011705 (PMC10729968; doi:10.1371/journal.pcbi.1011705)
Supplement: Attachment — Submitted filename: PLoS_Comp_Bio_ReviewResponse_round2.pdf [file pcbi.1011705.s019.pdf]

November 13th, 2023

Dear Christos and Zhaolei,

Thank you for taking the time to review our manuscript. We have addressed the remaining comment from the referee. We hope you find the revised manuscript suitable for publication.

Regards,

Zeqian Li, Ahmed Selim, Seppe Kuehn.

Comments to the Authors:
Please note here if the review is uploaded as an attachment.

Reviewer #1: Reviewer #1: I feel obligated to push back on the manner in which the prior expectation of correlation with phylogeny was discussed. The manuscript now includes these sentences: "It is important to recognize that the correlation between phylogeny and metabolic traits is known and has been recognized clinically and in the lab as an approach to identifying taxa from metabolic profiling [29,30]. However, our results provide a quantitative route to making such predictions, potentially providing new insights in other applications such as the human gut microbiome, where large libraries of isolates are available [31]. "

The first thing I have is that this statement give an incorrect impression. This is not the first paper to make a quantitative connection between phylogeny and metabolic traits. Here are some examples:

https://doi.org/10.1038/ismej.2012.160

https://doi.org/10.1038/nbt.2676

[These are just two such papers in a broad sea of literature]

The second thing is that this nod to the literature is coming rather late in the paper, whereas one would expect it in the introduction

We would like to thank the referee for pointing this out and for asking for a second look at the revisions to remedy this issue. Indeed we agree that our paper doesn't establish this phylogeny-trait correlation by a long shot and that this needs to be clear early in the manuscript. We have made the following revisions to place this point in the foreground:

- Abstract: added a line acknowledging the previous work on the phylogeny-trait correlation.
- Line 53: added acknowledgment of the phylogeny-trait correlation in previous work, including citations to the papers brought up by the referee.
- Line 221: changed language on the discussion of the phylogeny-trait correlation.

We hope these revisions make the point clear to the reader. Thanks for taking the extra time.
